# Geographic Variation in Private Equity Penetration Across Select Office-Based Physician Specialties in the US

**DOI:** 10.1001/jamahealthforum.2022.0825

**Published:** 2022-04-29

**Authors:** Yashaswini Singh, Jane M. Zhu, Daniel Polsky, Zirui Song

**Affiliations:** 1 Bloomberg School of Public Health, Department of Health Policy and Management, Johns Hopkins University, Baltimore, Maryland; 2 Division of General Internal Medicine, Oregon Health & Science University, Portland; 3 Department of Health Care Policy, Harvard Medical School, Boston, Massachusetts

## Abstract

This cross-sectional study examines geographic variations in private equity firm acquisitions of US physician practices across 6 specialties.

## Introduction

Little is known about the extent of physician practice acquisition by private equity (PE) firms. Private equity acquisition of US physician practices increased from 59 deals representing 843 physicians in 2013 to 136 deals representing 1882 physicians in 2016.^1^ Because PE acquisitions are not evenly distributed across the US, some markets are more affected. This cross-sectional study examined geographic variations in PE penetration of US physician practices (share of physicians in PE-acquired practices) across 6 specialties: dermatology, gastroenterology, ophthalmology, obstetrics/gynecology, orthopedics, and urology.

## Methods

We examined 2019 data from the IQVIA OneKey database, which provides physician and practice information on 9.7 million health professionals. These data are compiled from the American Medical Association (AMA) Physician Masterfile and proprietary data collection and are used to examine physician consolidation. This study followed the STROBE reporting guideline and was approved by the Oregon Health & Science University institutional review board, with a waiver of informed consent because this was non–human participant research.

Physician practices acquired by PE firms were defined by linking practice location information in OneKey to names of PE-acquired corporate owners identified in peer-reviewed research on PE acquisitions.^2,3,4,5^ Manual linkages were supplemented with internet searches, conducted in November 2021, of press releases and industry reports that identified practice acquisitions.

Private equity penetration was calculated at the hospital referral region (HRR) and state level as the estimated percentage of physicians across the 6 specialties who belonged to PE-acquired practices. The denominator reflects physicians in the evaluated specialties, identified using OneKey and verified against AMA data. If a physician appeared in multiple practices in an HRR, that physician was counted once. No physician appeared in multiple HRRs. We used 1-way analysis of variance to compare PE penetration across specialties, with significance at 2-sided *P* < .05. Stata, version 16.1 was used for analysis.

## Results

In 2019, 97 094 physicians practiced in the 6 specialties; of these, 4738 (4.9%) worked in PE-acquired practices. Private equity penetration was highest in dermatology (7.5% [851 of 11 324]), followed by gastroenterology (7.4% [845 of 11 484]), urology (6.5% [492 of 7609]), ophthalmology (5.1% [741 of 14 493]), obstetrics/gynecology (4.7% [1352 of 28 493]), and orthopedics (1.9% [460 of 23 891]).

**Figure 1.**
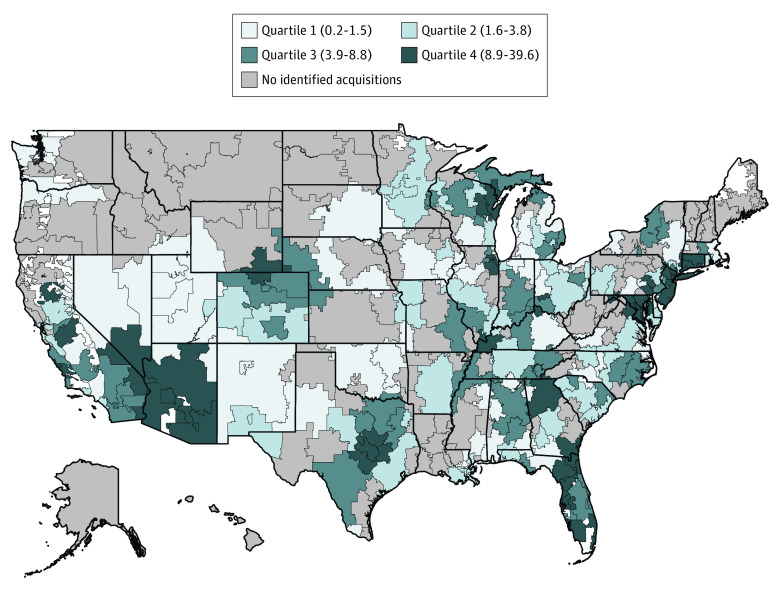
Private Equity (PE) Penetration Across 6 Office-Based Specialties by Hospital Referral Region (HRR) Private equity penetration was estimated at the HRR level and represents the mean share of physicians who were affiliated with PE-acquired practices across 6 specialties. The numerator included physicians (doctors of medicine [MDs] and doctors of osteopathic medicine [DOs]) in PE-acquired practices across the evaluated specialties. The denominator included physicians (MDs and DOs) across the evaluated specialties. Quartiles were determined by examining the distribution of PE penetration at the HRR level among 200 HRRs with any PE penetration. Numbers in parentheses are percentages and represent the range for each quartile.

Figure 1 shows geographic variation in PE penetration across specialties. Among 200 HRRs with PE penetration, a mean (SD) of 5.6% (6.3%) physicians were in PE-acquired practices. Private equity penetration was highest in the Northeast (6.8% [1270 of 18 708]) and lowest in the Midwest (3.8% [638 of 16 613]). Twelve states and Washington, DC, had an above-average share of physicians in PE practices; 11 states had no identified acquisitions. Washington, DC (18.2% [188 of 1031]), Arizona (17.5% [326 of 1866]), New Jersey (13.6% [464 of 3409]), Maryland (13.1% [195 of 1488]), Connecticut (12.6% [212 of 1688]), and Florida (10.8% [741 of 6852]) had the highest PE penetration.

**Figure 2.**
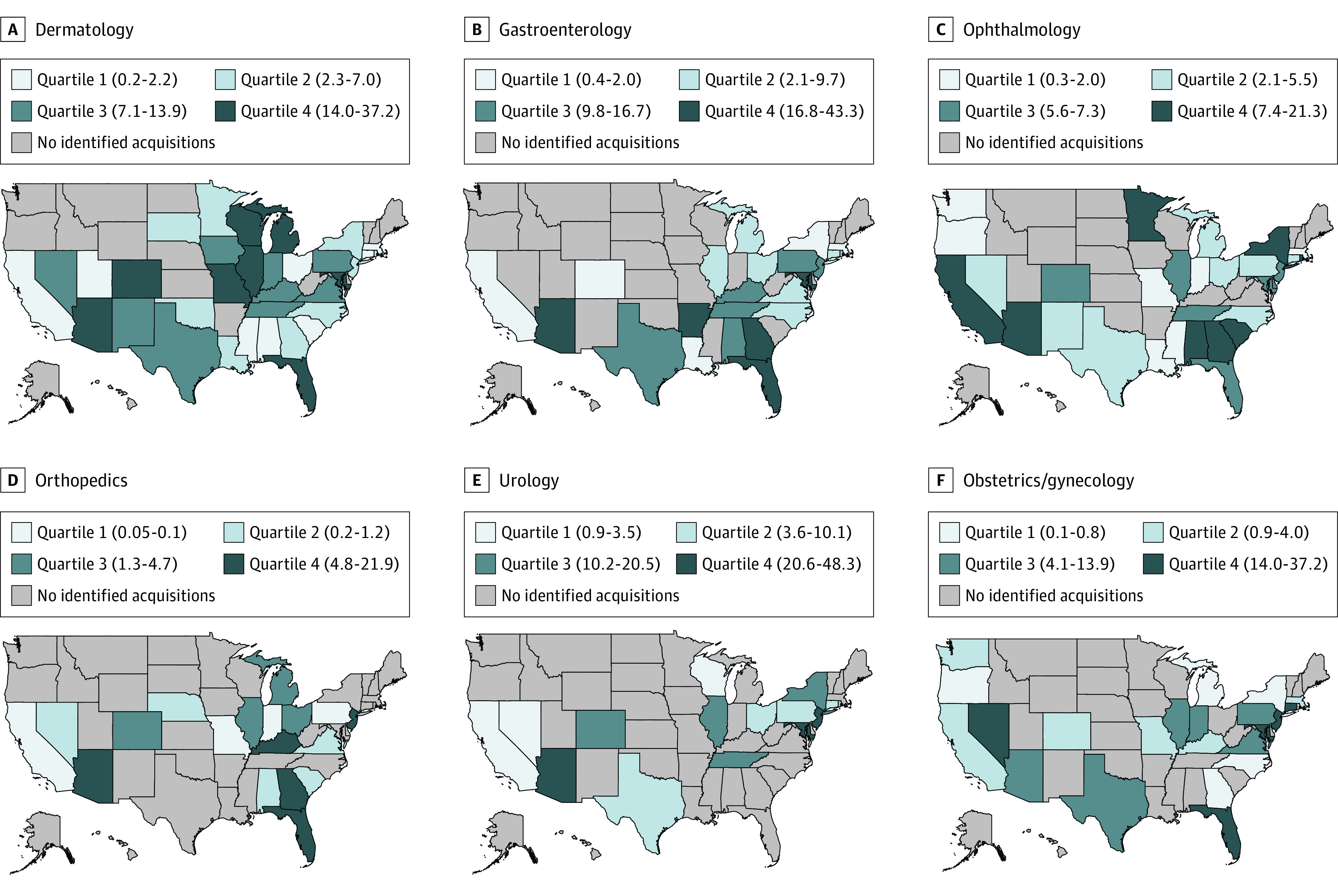
Geographic Variation in Private Equity (PE) Penetration by Physician Specialty and State Private equity penetration was estimated at the state level and represents the mean share of physicians who were affiliated with PE-acquired practices in each specialty. The numerator included physicians (doctors of medicine [MDs] and doctors of osteopathic medicine [DOs]) in PE-acquired practices in each specialty. The denominator included all physicians (MDs and DOs) in each specialty. Quartiles were determined by examining the distribution of PE penetration at the state level among states with any PE penetration. Numbers in parentheses are percentages and represent the range for each quartile.

Figure 2 shows the heterogeneity in PE penetration within specialties (*F* = 2.27; *P* = .047). Arizona was the only state with PE penetration in the top 2 quartiles in each specialty.

## Discussion

Across examined specialties, PE acquisitions of US physician practices were concentrated in certain HRRs in the Northeast, Florida, and Arizona. Because some PE acquisitions consolidate physician practices into larger organizations, geographic concentration of PE penetration may be associated with reduced physician competition, which could lead to increased prices. If PE acquisitions induce practice consolidation among remaining independent practices with financial pressures, this spillover effect may further hinder competition, underscoring the importance of monitoring practice consolidation and the ownership and regulatory environment of acquisitions. Because many PE-acquired hospitals were located in similar regions, joint PE penetration into hospital and physician markets deserves additional study.

Limitations include that data may not have captured all PE acquisitions; thus, acquisitions may be underestimated, although our dermatology estimates are consistent with a previous study.^6^ Use of secondary data from OneKey may include sampling and measurement error, although our estimates are consistent with AMA data.
